# Parturition failure in mice lacking *Mamld1*

**DOI:** 10.1038/srep14705

**Published:** 2015-10-05

**Authors:** Mami Miyado, Kenji Miyado, Momori Katsumi, Kazuki Saito, Akihiro Nakamura, Daizou Shihara, Tsutomu Ogata, Maki Fukami

**Affiliations:** 1 Department of Molecular Endocrinology, National Research Institute of Child Health and Development, Tokyo 157-8535, Japan; 2 Department of Reproductive Biology, National Research Institute of Child Health and Development, Tokyo 157-8535, Japan; 3 Department of Pediatrics, Hamamatsu University School of Medicine, Hamamatsu 431-3192, Japan

## Abstract

In mice, the onset of parturition is triggered by a rapid decline in circulating progesterone. Progesterone withdrawal occurs as a result of functional luteolysis, which is characterized by an increase in the enzymatic activity of 20α-hydroxysteroid dehydrogenase (20α-HSD) in the corpus luteum and is mediated by the prostaglandin F2α (PGF_2α_) signaling. Here, we report that the genetic knockout (KO) of *Mamld1*, which encodes a putative non-DNA-binding regulator of testicular steroidogenesis, caused defective functional luteolysis and subsequent parturition failure and neonatal deaths. Progesterone receptor inhibition induced the onset of parturition in pregnant KO mice, and MAMLD1 regulated the expression of *Akr1c18*, the gene encoding 20α-HSD, in cultured cells. Ovaries of KO mice at late gestation were morphologically unremarkable; however, *Akr1c18* expression was reduced and expression of its suppressor *Stat5b* was markedly increased. Several other genes including *Prlr*, *Cyp19a1*, *Oxtr*, and *Lgals3* were also dysregulated in the KO ovaries, whereas PGF_2α_ signaling genes remained unaffected. These results highlight the role of MAMLD1 in labour initiation. MAMLD1 likely participates in functional luteolysis by regulating *Stat5b* and other genes, independent of the PGF_2α_ signaling pathway.

In most mammals including mice, uterine quiescence during pregnancy is maintained by circulating progesterone synthesized primarily in the ovarian luteal cells^1^,^2^. Progesterone binds to its receptor in the uterus and suppresses the expression of genes involved in myometrial contraction^3^,^4^. Previous studies have shown that signal transducer and activator of transcription 5b (STAT5B) is essential to sustain blood progesterone levels in pregnant mice^5^,^6^,^7^. STAT5B inhibits ovarian expression of *Akr1c18*, the gene for 20α-hydroxysteroid dehydrogenase (20α-HSD) that converts progesterone into an inactive metabolite 20α-hydroxyprogesterone (20α-OHP)^5^. From 18 days post coitum (dpc), *i.e*., 24–36 hours before term, progesterone secretion from the ovary progressively declines through processes referred to as functional and structural luteolysis^1^,^5^. Functional luteolysis is an enzymatic shift characterized by an increase in 20α-HSD activity^1^,^5^. This process is followed by structural luteolysis, in which the corpus luteum undergoes morphological changes and cellular apoptosis^1^,^8^. To date, multiple molecules have been implicated in functional luteolysis^1^. Of these, prostaglandin F2α (PGF_2α_) upregulates *Akr1c18* via a signaling pathway consisting of PGF_2α_, PGF_2α_ receptor (FP), JUND, and nuclear receptor subfamily 4 group A member 1 (NR4A1, also known as NUR77)^1^,^9^,^10^. The Gq/11 protein family also serves as a component of the PGF_2α_ signaling pathway^11^. Genetic knockout (KO) of *Akr1c18*, *Fp*, or *Gα*_*q/11*_ leads to persistent progesterone production and subsequent parturition failure^5^,^11^,^12^,^13^. In addition, *Fp* KO perturbs expression of several steroidogenic genes in the corpus luteum, which may also be relevant to delayed parturition^14^. Other factors, including NOTCH 1 and 4, oxytocin receptor (OXTR), and galectin 3, also participate in luteolytic processes and/or regulation of *Akr1c18*^1^,^15^,^16^,^17^; however, it remains unknown whether STAT5B plays a role in functional luteolysis.

*MAMLD1* on the human X chromosome (NM_001177465) is a causative gene for disorders of sex development in 46,XY individuals^18^. Loss-of-function mutations in *MAMLD1* have been identified in male patients with hypospadias^18^,^19^,^20^. Murine *Mamld1* (NM_001081354) also resides on the X chromosome and is strongly expressed in the Leydig and Sertoli cells of the fetal testis^18^. *In vitro* knockdown assays using mouse Leydig tumour cells (MLTC1) and *in vivo* analysis of male *Mamld1* KO mice indicated that MAMLD1 transactivates several Leydig cell-specific genes including *Star*, *Cyp11a1*, *Cyp17a1*, *Hsd3b1*, and *Insl3* without exerting a demonstrable DNA-binding capacity^21^,^22^,^23^. While male *Mamld1* KO mice showed no hypospadias, the phenotypic difference between human patients and KO mice was explicable by species differences in the process of male sex development^23^. To date, the function of MAMLD1 in females has not been investigated, although previous analyses detected strong expression of *Mamld1* in the ovaries of adult mice^18^. In the present study, we analyzed phenotypic and molecular characteristics of female *Mamld1* KO mice.

## Results

### *Mamld1* KO causes parturition failure in female mice

Prior to this study, we generated a mouse strain in which the genomic structure of *Mamld1* was disrupted by substituting a *PGK-neo* cassette for *Mamld1* exon 3 that corresponds to approximately two-thirds of the coding region^23^. We have reported that male *Mamld1* KO mice retained normal external genitalia and fertility, despite having mildly impaired expression of Leydig cell-specific genes in the fetal testis^23^.

In this study, we analyzed the phenotype of female *Mamld1* KO mice. The mice were healthy and exhibited no discernible anomalies. Furthermore, the mice were fertile when mated with male wildtype (WT) or *Mamld1* KO mice. However, female KO mice frequently showed delayed parturition (Table 1). More than 50% of KO mice gave birth to their first pups at 20.5 dpc or later, while approximately 80% of WT animals gave birth at 19.5 dpc. The frequency of delayed parturition (≥20.5 dpc) in WT animals was comparable between this study and previous studies^3^,^24^. The genotype of the mated male mice (WT or KO) had no influence on the parturition timing of the female WT or KO mice.

### Pups born to *Mamld1* KO mothers have a high neonatal mortality rate and can be rescued by caesarean operation

**Figure 1:**
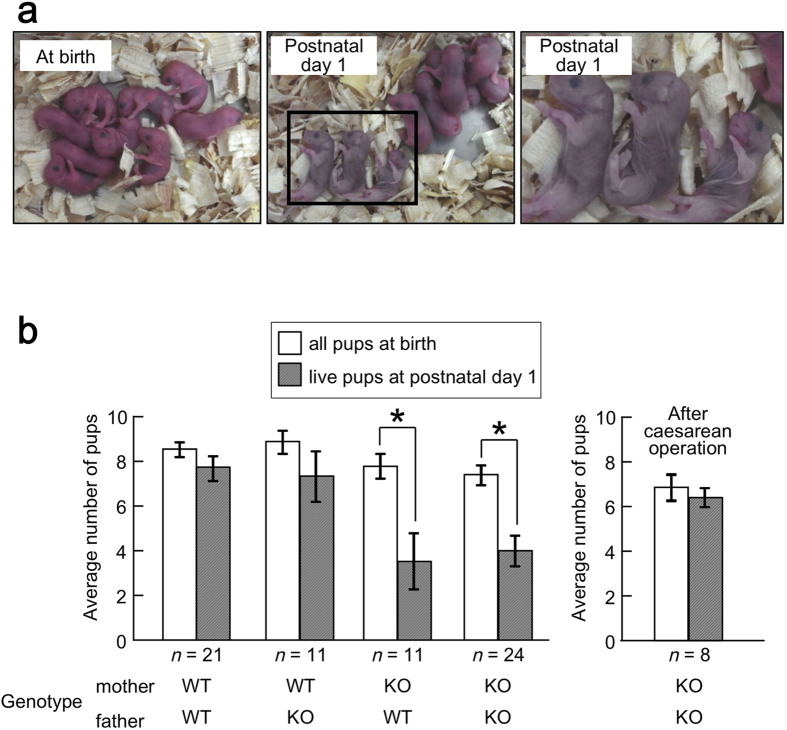
Phenotypes of pups born to *Mamld1* knockout (KO) mothers. (**a**) Pups born to KO mothers at 20.5 dpc. Pups showed a high neonatal mortality, although they had no congenital anomalies. The photographs were taken by M.M. and M.F. at the National Research Institute for Child Health and Development. (**b**) Average number of births (white bars) and that of live pups at postnatal day 1 (gray bars). Pups born to KO mothers showed a significantly higher newborn mortality rate than those born to wildtype (WT) mothers (asterisks). Paternal genotype had no effect on the number of pups. Frequent newborn deaths were eliminated by caesarean operation. The results are expressed as the mean ± SEM.

We examined the number of pups born to WT and *Mamld1* KO mothers. Although the average number of pups at birth was comparable between the two groups, the average number of pups alive at postnatal day 1 was significantly lower in KO mothers (Fig. 1a,b). Approximately half of the pups born to *Mamld1* KO mothers died within the first 24 hours after birth, while >80% of pups born to WT mothers survived beyond this period. The dead pups of KO mothers exhibited no apparent malformations (Fig. 1a). Most pups survived beyond postnatal day 1 remained alive until adulthood. The newborn mortality rates of WT and KO mothers were not affected by paternal genotype (WT or KO). The sex ratio of the dead pups was almost 1:1. Thus, the neonatal deaths were more likely the result of an aberrant maternal condition rather than inborn defects in the pups.

It is known that parturition failure in female *Fp* KO mice results in frequent fetal death^12^,^13^. To clarify whether the high mortality rate of pups born to *Mamld1* KO mothers was due to delayed parturition, we performed caesarean operations on the day of the expected term (19.5 dpc). The operations significantly improved the survival rate of pups; at postnatal day 1, the average number of live pups born to the operated KO mothers was comparable to that born to non-operated WT mothers (Fig. 1b).

### Progesterone withdrawal is impaired in pregnant *Mamld1* KO mice

Previous studies have shown that parturition failure is caused by defects in functional luteolysis that lead to persistent progesterone production^5^,^12^,^13^,^15^,^24^; however, it can also be caused by uterine lesions such as defective myometrial contraction or delayed cervical ripening^25^,^26^. To determine whether progesterone withdrawal is impaired in pregnant *Mamld1* KO mice, we measured serum levels of progesterone and other steroids. In this study, we utilized liquid chromatography tandem mass spectrometry (LC-MS/MS), which is more sensitive and accurate than conventional immunoassays^27^. Serum samples were collected from pregnant WT and KO mice at 18.5 dpc, a stage at which circulating progesterone usually declines in WT mice^25^. Serum progesterone was significantly higher in KO mice than in WT animals (Table 2). In contrast, serum levels of 20α-OHP, the inactive metabolite of progesterone, remained low in KO mice. Altered serum levels of progesterone and 20α-OHP were also observed in KO mice at 20.5 dpc (Table 2). Blood levels of testosterone and estradiol were comparable between WT and KO mice.

To confirm that impaired progesterone withdrawal is the major cause of parturition failure in *Mamld1* KO mice, we treated pregnant mice with the progesterone receptor antagonist RU486. Administration of 150 μg RU486 at 17.5 or 18.5 dpc invariably induced vaginal bleeding (the signs of labour initiation) and/or delivery of a pup(s) within 24 hours in both WT and KO mice (Supplementary Table S1).

**Figure 2:**
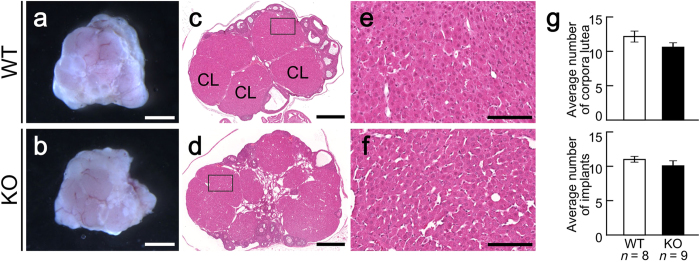
Morphological analysis. (**a–f**) Morphological findings of the ovaries obtained from pregnant WT and *Mamld1* KO mice at 18.5 dpc. Scale bars: 1 mm (**a,b**), 500 μm (**c,d**), and 100 μm (**e,f**). CL, corpus luteum. (**g**) Average number of corpora lutea in the ovary (upper panel) and that of implants in the uterus (lower panel) at 18.5 dpc. The results are expressed as the mean ± SEM.

We also examined whether *Mamld1* KO affects ovarian structures. The size and appearance of the ovaries were comparable between pregnant WT and KO mice at 18.5 dpc (Fig. 2a,b). No apparent histological changes were observed in the ovaries of KO mice (Fig. 2c–f). Furthermore, the average number of corpora lutea in the ovary and that of implants in the uterus were similar between WT and KO mice (Fig. 2g). The position of uterine implantation was also normal in KO mice. These data indicate that *Mamld1* KO exerts a deleterious effect on functional luteolysis, but not on ovary development, ovulation, luteinization or implantation.

### In ovaries of WT mice during late gestation, *Mamld1* is continuously expressed, while expression levels of *Akr1c18, Nr4a1,* and *Stat5b* drastically change after 17.5 dpc

**Figure 3:**
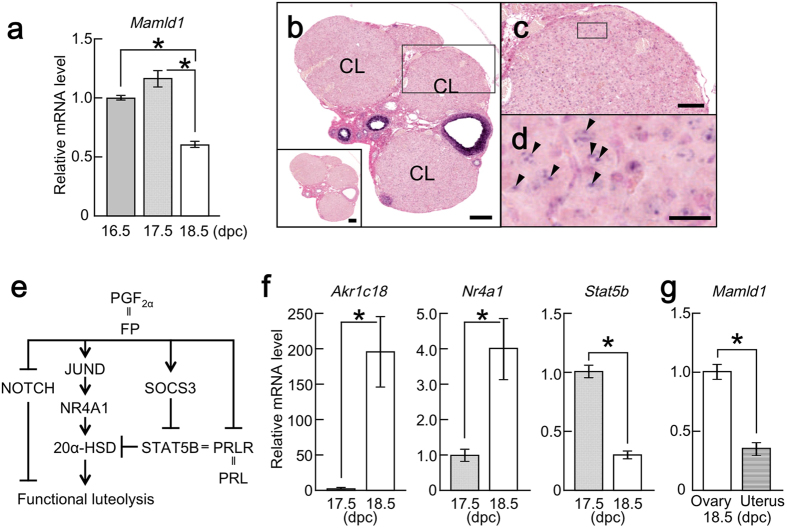
*Mamld1* expression in pregnant WT mice. (**a**) *Mamld1* expression in whole ovaries from pregnant WT mice at 16.5 (*n* = 3), 17.5 (*n* = 5), and 18.5 dpc (*n* = 8). mRNA levels relative to that of *Gapdh* are shown. The results are expressed as the mean ± SEM. The average of mRNA levels at 16.5 dpc was defined as 1.0. Asterisks indicate statistical significance. (**b–d**) *Mamld1* expression in corpora lutea and follicles. Arrowheads indicate *Mamld1* signals in corpus luteum. Scale bars: 200 μm (**b**), 100 μm (**c**), and 20 μm (**d**). No specific expression of the negative control (a sense probe). CL, corpus luteum. (**e**) Known factors involved in functional luteolysis. Arrow and bar headed lines indicate stimulatory and inhibitory effects, respectively. Double lines indicate protein-receptor bindings. FP, prostaglandin F2α receptor; PRL, prolactin; PRLR, PRL receptor. (**f**) Gene expression in the whole ovaries in pregnant WT mice at 17.5 and 18.5 dpc (*n* = 5 and 8, respectively). The average of mRNA levels at 17.5 dpc was defined as 1.0. (**g**) *Mamld1* expression in the whole ovaries (Ovary, *n* = 8) and uteri (Uterus, *n* = 4) from pregnant WT mice at 18.5 dpc. The average of mRNA levels in the whole ovaries was defined as 1.0.

We examined *Mamld1* expression in the ovaries of WT mice at late gestation. Real-time PCR detected continuous expression in the ovaries, with the highest expression at 17.5 dpc (Fig. 3a). *In situ* hybridization of the murine ovary at 18.5 dpc showed clear signals for *Mamld1* mRNA in the corpora lutea as well as in the primary, secondary, vesicular, and mature follicles (Fig. 3b–d).

We also analyzed mRNA levels of *Akr1c18*, *Nr4a1*, and *Stat5b* in ovaries of pregnant WT mice at 17.5 and 18.5 dpc. These genes showed drastic changes in expression between 17.5 and 18.5 dpc, as reported previously^10^. *Akr1c18* and *Nr4a1* expression was significantly higher at 18.5 dpc than at 17.5 dpc, while *Stat5b* expression was markedly decreased at 18.5 dpc (Fig. 3f).

In addition, we analyzed *Mamld1* expression in the uteri of pregnant WT mice at 18.5 dpc. A relatively weak expression was detected in the uteri, as compared to that in the ovaries (Fig. 3g).

### MAMLD1 regulates *Akr1c18* expression *in vivo* and *in vitro*

**Figure 4:**
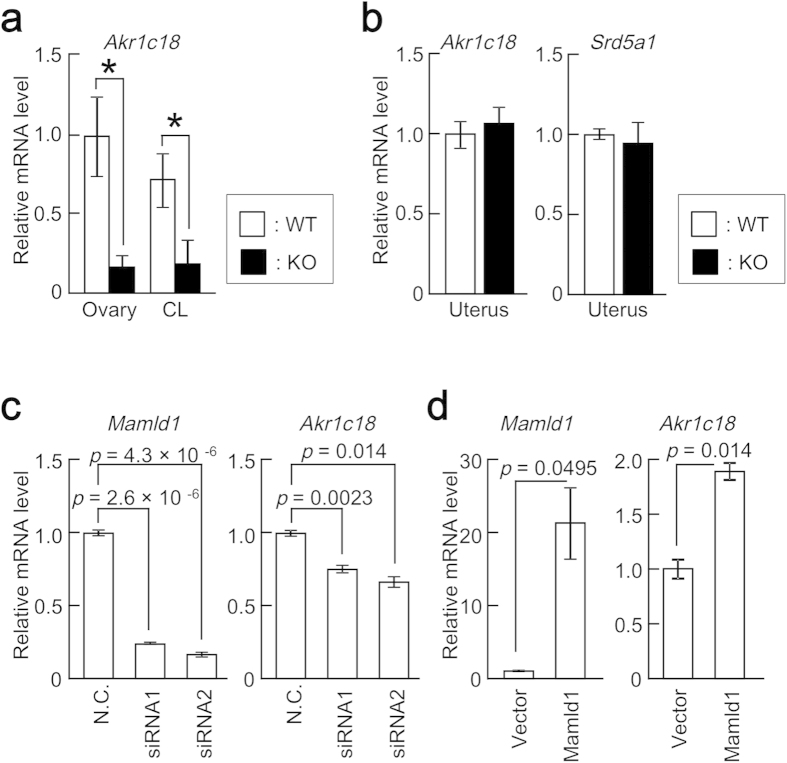
The effect of MAMLD1 on *Akr1c18* expression. (**a**) *Akr1c18* expression in the whole ovaries (Ovary) and corpora lutea (CL) of pregnant WT (*n* = 8, white bars) and *Mamld1* KO (*n* = 9, black bars) mice at 18.5 dpc. mRNA levels relative to that of *Gapdh* are shown. The results are expressed as the mean ± SEM. The average of mRNA levels in the whole ovaries of WT mice was defined as 1.0. Significant differences between WT and KO animals are indicated by asterisks. (**b**) *Akr1c18* and *Srd5a1* expression in the uteri (Uterus) of pregnant WT (*n* = 4, white bars) and KO (*n* = 4, black bars) mice at 18.5 dpc. The average of mRNA levels in WT mice was defined as 1.0. (**c**) *Mamld1* knockdown assays. N.C., negative control (non-targeting siRNA). The average of mRNA levels in N.C. was defined as 1.0. (**d**) Overexpression experiments of *Mamld1*. Vector, empty expression vector. The average of mRNA levels in Vector was defined as 1.0.

We examined the expression of *Akr1c18*/20α-HSD in pregnant WT and *Mamld1* KO mice at 18.5 dpc. Real-time PCR analysis showed significantly decreased *Akr1c18* expression in the whole ovaries and corpora lutea of KO mice (Fig. 4a), and Western blot analysis confirmed the reduction of 20α-HSD protein expression in the ovaries of KO mice (Supplementary Fig. S1). In contrast, mRNA levels of *Akr1c18* in the uteri were comparable between WT and KO mice at 18.5 dpc (Fig. 4b). Expression of *Srd5a1* for steroid 5α reductase, which mediates local progesterone metabolism in the uterus, remained unaffected in KO mice (Fig. 4b). *Akr1c18* expression remained low in the KO mice at 20.5 dpc (Supplementary Fig. S2).

To confirm the effect of MAMLD1 on *Akr1c18* expression, we performed *in vitro* assays. In these experiments, we used MLTC1, which has high endogenous expression of both *Mamld1* and *Akr1c18*. First, we carried out knockdown assays using two siRNAs for *Mamld1*. When *Mamld1* mRNA levels were suppressed to ~25% by the siRNAs, *Akr1c18* mRNA levels were reduced to ~75% (Fig. 4c). Next, we performed *Mamld1* overexpression experiments. Transient transfection with a *Mamld1* expression vector resulted in a ~2-fold increase of *Akr1c18* mRNA after a 24-hour cell culture (Fig. 4d).

### *Mamld1* KO dysregulates *Stat5b* and other genes in the ovaries of pregnant mice

**Figure 5:**
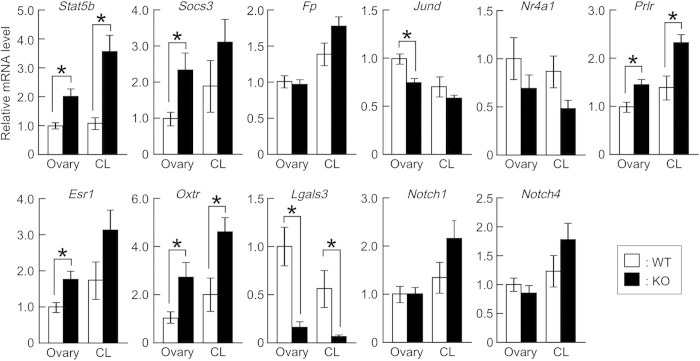
Expression patterns of functional luteolysis-related genes in pregnant WT and *Mamld1* KO mice. Relative mRNA levels of genes in the whole ovaries (Ovary) and corpora lutea (CL) in pregnant WT (*n* = 6, white bars) and KO (*n* = 6, black bars) mice at 18.5 dpc are shown. The results are expressed as the mean ± SEM. The average of mRNA levels in the whole ovaries of WT mice was defined as 1.0. Asterisks indicate statistical significance.

We examined gene expression patterns in the whole ovaries and corpora lutea of pregnant WT and *Mamld1* KO mice at 18.5 dpc (Fig. 5). The most remarkable changes in KO mice were the significantly increased mRNA levels of *Stat5b*, despite overexpression of *Socs3*, which encodes a putative inhibitor of *Stat5*. *Prlr* and *Esr1* were also upregulated. In contrast, *Fp*, *Jund*, and *Nr4a1* were not affected, except for a slightly decreased expression of *Jund* in the whole ovaries. Increased levels of STAT5B protein and unaffected levels of NR4A1 protein in KO mice ovaries were confirmed by Western blot analysis (Supplementary Fig. S1). Markedly increased *Stat5b* mRNA expression was also observed in pregnant KO mice at 20.5 dpc (Supplementary Fig. S2).

**Figure 6:**
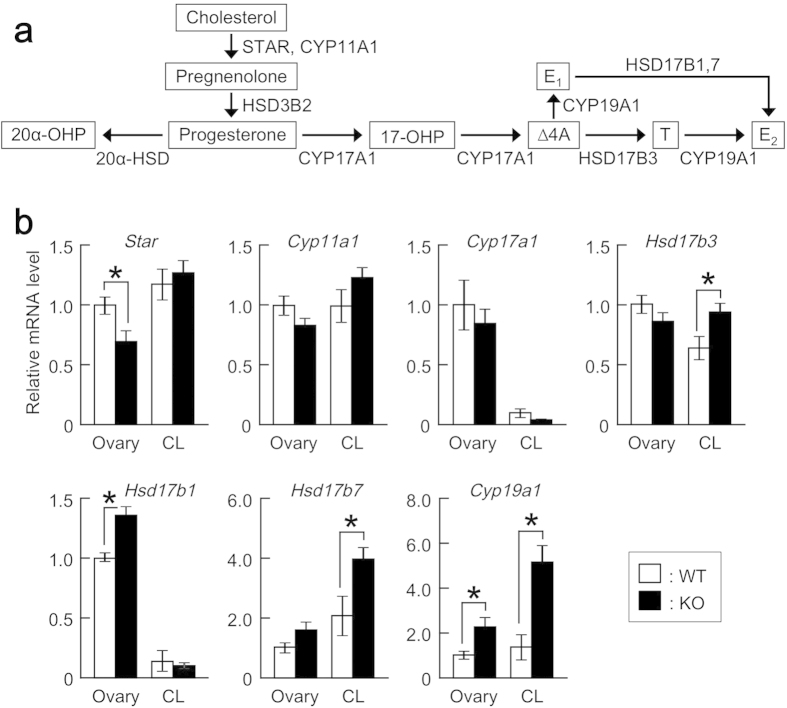
Expression patterns of steroidogenic genes in pregnant WT and *Mamld1* KO mice. (**a**) Enzymes involved in the steroidogenic pathway. 17-OHP, 17-hydroxyprogesterone; 20α-OHP, 20α-hydroxyprogesterone; E_1_, estrone; Δ4A, androstenedione; T, testosterone; E_2_, estradiol. (**b**) Gene expression in the whole ovaries (Ovary) and corpora lutea (CL) in pregnant WT (*n* = 6, white bars) and KO (*n* = 6, black bars) mice at 18.5 dpc. mRNA levels relative to that of *Gapdh* are shown. The results are expressed as the mean ± SEM. The average of mRNA levels in the whole ovaries of WT mice was defined as 1.0. Significant differences between WT and KO animals are indicated by asterisks. *Hsd3b2* was undetectable in both WT and KO mice.

We also analyzed mRNA levels of other genes involved in ovarian steroidogenesis and in the luteolytic processes (Figs 5 and 6). Gene expression patterns were grossly similar in the whole ovaries and corpora lutea. Among the steroidogenic genes, *Cyp19a1* was significantly upregulated. Expression levels of *Hsd17b3*, *Hsd17b1*, and *Hsd17b7* were mildly increased, while mRNA levels of *Cyp11a1* and *Cyp17a1* remained unchanged. *Star* expression was slightly decreased, but only in the whole ovaries. Of the genes involved in the luteolytic processes, *Oxtr* was upregulated, while *Lgals3* encoding anti-apoptotic factor galectin 3 was downregulated. *Notch 1* and *4* were unaffected.

## Discussion

Targeted deletion of *Mamld1* in female mice caused parturition failure and frequent neonatal deaths without affecting ovarian morphology. This phenotype likely results from attenuated functional luteolysis, because expression of *Akr1c18* mRNA and 20α-HSD protein was markedly decreased in the ovaries of pregnant *Mamld1* KO mice at 18.5 dpc. Consistent with this, ratios of 20α-OHP to progesterone in blood samples were lower in KO mice than in WT animals. Although the serum levels of progesterone and 20α-OHP in our mice differed from those in previous reports^5^,^10^, this can be ascribed to the difference in the methods (LC-MS/MS vs. conventional immunoassays) and sampling points (the day when a vaginal plug was observed was designated as 0.5 dpc in this study and as 1.0 dpc in previous studies). Attenuated functional luteolysis seemed to persist in KO mice after the day of the expected term. We found that inhibition of progesterone signaling by RU486 induced vaginal bleeding (the signs of labour initiation) and/or delivery of a pup(s) in KO mice. *In vitro* assays indicated that MAMLD1 upregulates *Akr1c18* in MLTC1, although these results need to be confirmed in further studies using cells of ovarian origin. While previous studies have shown that local progesterone metabolism in the uterus can also affect parturition timing^25^,^26^, mRNA levels of *Akr1c18* and *Srd5a1* in the uteri remained unaffected in *Mamld1* KO mice. Furthermore, *Mamld1* was continuously expressed in the ovaries during late gestation, and only weakly expressed in the uteri. Collectively, the results suggest that MAMLD1 is involved in upregulation of *Akr1c18* in ovaries of pregnant mice at late gestation.

The phenotype of pregnant *Mamld1* KO mice overlaps with that of *Fp* KO mice^12^,^13^; however, expression of the PGF_2α_ signaling pathway genes, *Fp*, *Jund*, and *Nr4a1*, was not significantly altered in the ovaries of *Mamld1* KO mice at 18.5 dpc. Likewise, protein expression of NR4A1, the most downstream component of the PGF_2α_ signaling pathway that directly binds to the *Akr1c18* promoter, remained unaffected in KO mice ovaries. Thus, the function of MAMLD1 appears to be independent of the PGF_2α_ signaling pathway, although mRNA expression of the Gq/11 protein family, a recently identified component of this pathway^11^, was not analyzed in the present study. In contrast, *Stat5b* and *Prlr* were markedly upregulated in KO mice ovaries. Increased *Prlr* expression can be ascribed to high STAT5B activity, which transactivates *Prlr*^28^. Likewise, *Esr1*, the potential target of STAT5B in rats^29^, was also upregulated in *Mamld1* KO mice. To date, STAT5B has not been implicated in functional luteolysis, although it suppresses *Akr1c18* during mid-gestation^5^. We confirmed that *Stat5b* expression significantly declined in pregnant WT mice ovaries after 17.5 dpc. Our data imply that *Stat5b* suppression mediated by MAMLD1 is critical for functional luteolysis. Since MAMLD1 protein transactivates various genes in the fetal testis without demonstrable DNA binding capacity^21^,^23^, MAMLD1 may regulate *Stat5b* expression as a non-DNA-binding co-activator. In this regard, it is noteworthy that the phenotypic severity of pregnant *Mamld1* KO mice was milder than that of *Fp* KO mice. While *Mamld1* KO permits a term delivery in approximately half of pregnant mice, *Fp* KO leads to parturition failure and loss of pups in all mice^12^,^13^. Likewise, the increase in blood progesterone levels at the end of pregnancy was less significant in *Mamld1* KO mice than in *Fp* KO mice. These results are consistent with the findings that *Akr1c18* mRNA levels in the ovaries were decreased by 70–80% in pregnant *Mamld1* KO mice, and by 100% in *Fp* KO mice^10^. This suggests that although MAMLD1 and PGF_2α_ signaling are essential for the luteolytic process, the role of MAMLD1 is relatively minor compared to that of PGF_2α_ signaling.

Several other genes were dysregulated in pregnant *Mamld1* KO mice ovaries. First, *Cyp19a1*, *Hsd17b3*, *Hsd17b1*, and *Hsd17b7* involved in ovarian steroidogenesis were upregulated. These molecular alterations did not affect blood sex hormone levels. However, perturbed steroidogenesis may play a role in parturition failure of *Mamld1* KO mice, because previous studies suggested that the androgen:estrogen synthesis ratio in the ovaries affects the luteolytic process^14^. Second, *Oxtr* expression was increased in the KO mice ovaries. It has been shown that administration of low-dose oxytocin results in persistent progesterone production and subsequent parturition failure, whereas high-dose oxytocin causes uterine contraction and early labour^12^. Since downregulation of *Oxtr* in the ovaries and its upregulation in the uteri were proposed to induce the onset of parturition^2^,^30^, elevated expression of *Oxtr* in the ovaries of *Mamld1* KO mice may be associated with delayed parturition. Third, expression of *Lgals3* was decreased in the whole ovaries and corpora lutea of KO mice. *Lgals3* is co-expressed with *Akr1c18* in the corpora lutea, and galectin 3 encoded by *Lgals3* contributes to the elimination of luteal cells^8^. Thus, decreased *Lgals3* expression in the ovaries of *Mamld1* KO mice may also be relevant to impaired luteolysis. Lastly, expression of *Notch 1* and *4* remained intact in KO mice. Thus, although MAMLD1 has sequence similarity with a Notch co-factor Mastermind-like 2^21^, the function of MAMLD1 in the ovaries is unlikely to be associated with Notch signals.

In summary, our results indicate that MAMLD1-mediated *Stat5b* suppression is essential for term delivery in mice. MAMLD1 appears to participate in a complex molecular network in the ovaries and regulate functional luteolysis, without affecting expression of PGF_2α_ signaling genes. This study provides novel insights into molecular mechanisms of mammalian reproduction.

## Methods

### Treatment of animals

Animal experiments in this study were approved by the Animal Care Committee at the National Research Institute for Child Health and Development (project number: A2008-001). All experiments were performed in accordance with the institutional guidelines of the care and use of laboratory animals. All mice were housed under specific pathogen-free controlled conditions with a 12-hour light-dark cycle. Food and water were available *ad libitum*.

### *Mamld1* KO mice

Male *Mamld1* KO mice were generated by targeting deletion of exon 3^23^. The mice were backcrossed with the C57BL/6N strain (Sankyo Labo Service Corp. Inc., Tokyo, Japan).

### Cross-mating and caesarean operation

Cross-mating was performed between female *Mamld1* KO mice and male WT or KO mice and between female WT mice and male WT or KO mice. Female mice from 7 to 25 weeks of age and male mice from 8 to 40 weeks of age were used for mating. The noon of the day when a vaginal plug was observed was designated as 0.5 dpc. Vaginal bleeding (the signs of labour initiation) or delivery of the first pup was defined as the onset of parturition. Caesarean operation was performed for *Mamld1* KO mice at 19.5 dpc. After birth, the pups were nursed by lactating WT animals.

### Measurement of serum steroid metabolites

Blood samples were collected from the right ventricle of the heart of euthanized pregnant WT and KO mice at 18.5 dpc, pregnant KO mice at 20.5 dpc, and WT mice at 0 or 1 day postpartum. The serum was separated by centrifugation and stored at −80 °C until hormone measurements were performed. Serum steroid metabolites were measured by LC-MS/MS (ASKA Pharma Medical, Kanagawa, Japan).

### Parturition induction by progesterone receptor antagonist

The progesterone receptor antagonist RU486 (mifepristone; Sigma-Aldrich, St. Louis, MO) was administered to pregnant mice at 17.5 or 18.5 dpc. One ml of solution containing 150 μg RU486 in 6% ethanol was subcutaneously injected in the bilateral hind legs.

### Morphological and quantitative analyses of corpora lutea and uterine implants

We analyzed the morphology of ovaries obtained from pregnant WT and KO mice at 18.5 dpc. Tissue samples were fixed with 4% paraformaldehyde, dehydrated, and embedded in paraffin. Serial 6 μm sections were mounted on microscope slides. The samples were stained with hematoxylin-eosin, and the number of corpora lutea in the ovary and implants in the uterus were counted under a stereoscope.

### Real-time RT-PCR analysis

Whole ovaries and corpora lutea were isolated from pregnant WT (*n* = 12–16) and *Mamld1* KO (*n* = 12–18) mice at 18.5 dpc, and uteri were isolated from four mice of each genotype at the same stage. Whole ovaries were also isolated from pregnant WT mice at 16.5 and 17.5 dpc (*n* = 3 and 5, respectively), pregnant KO mice at 20.5 dpc (*n* = 5), and WT mice at 0 or 1 day postpartum (*n* = 4). Tissues were immediately soaked in RNAlater solution (Life Technologies, Carlsbad, CA). Total RNA was extracted from homogenized samples by ISOGEN (Nippongene, Tokyo, Japan) and RNeasy Kit (QIAGEN, Valencia, CA). Contaminated genomic DNA was removed with a TURBO DNA-free kit (Life Technologies). cDNA was synthesized from 200 ng total RNA using a High Capacity cDNA Reverse Transcription kit (Life Technologies). We measured relative mRNA levels of genes implicated in the luteolytic process and/or regulation of *Akr1c18*. *Gapdh* was used as an internal control. The assays were performed using the ABI 7500 Fast real-time PCR system and TaqMan gene expression assay kit (Life Technologies). Primers and probes used in this study are listed in Supplementary Table S2.

### *In situ* hybridization

We examined *Mamld1* expression in the ovaries obtained from pregnant WT mice at 18.5 dpc. Paraffin sections were prepared as described above. *In situ* hybridization was performed using an antisense RNA probe for mouse *Mamld1*^18^ (Genostaff Inc., Tokyo, Japan). The probe was digoxigenin-labeled using DIG RNA Labeling Mix (Roche, Basel, Switzerland). A sense cRNA for mouse *Mamld1* was used as a negative control. The colour of the probes was developed with NBT/BCIP solution (Sigma-Aldrich) and the sections were counterstained with Kernechtrot solution (Mutoh Chemical, Tokyo, Japan).

### Western blot analysis

Tissue extracts were prepared from the ovaries of pregnant mice at 18.5 dpc and separated by standard SDS-PAGE (7.5% or 4–20% gradient gel; Bio-Rad, Hercules, CA). PVDF membranes were incubated in the solution containing the primary antibody. We used anti-20α-HSD antibodies (EB4002; KeraFAST Inc., Boston, MA), anti-NR4A1 antibodies (ab13851; Abcam, Cambridge, MA), and anti-STAT5B antibodies (ab178941; Abcam). Anti-ACTIN antibodies (A2066; Sigma-Aldrich) were used as an internal control. The signals were detected using Clarity Western ECL Substrate (Bio-Rad). All analyses were performed using three independent samples per group.

### *In vitro* functional assays

MLTC1 (CRL-2065^TM^; ATCC, Manassas, VA) were maintained in RPMI 1640 medium containing 10% fetal bovine serum. For *Mamld1* knockdown assays, the cells were seeded in 6-well plates (1.0 × 10^5^ cells/well) and transiently transfected with two siRNAs, i.e., siRNA1 (sense: 5′-CAGGAAUCGGGAACCAGUAAGAGAA-3′; and anti-sense: 5′-UUCUCUUACUGGUUCCCGAUUCCUG-3′) and siRNA2 (sense: 5′-CAGAGAUGCAGAUGCCCACAUUAAA-3′; and anti-sense: 5′-UUUAAUGUGGGCAUCUGCAUCUCUG-3′), or with a non-targeting control RNA (4611G; Life Technologies) (20 nM final concentration), using Lipofectamine RNAiMAX (Life Technologies). For *Mamld1* overexpression assays, the cells were seeded in 12-well plates (1.0 × 10^5^ cells/well) and transfected with 200  ng of the expression vector of *Mamld1* or an empty expression vector (pCMV-Myc vector; Takara Bio, Otsu, Japan), using Lipofectamine 3000 (Life Technologies). The full-length *Mamld1* cDNA, which contains 2,412 nucleotides corresponding to the coding region without both 5′- and 3′-untranslated regions, was amplified from mouse fetal testis-derived cDNA mixture (C57BL/6N; Sankyo Labo Service Corp. Inc.), and subcloned into a plasmid that was included in the TOPO TA cloning kit (Life Technologies). The cDNA that was missing the start codon was then subcloned into a pCMV-Myc vector to construct the *Mamld1* expression vector.

 The cells were harvested 24 hours after transfection. Total RNA were subjected to cDNA synthesis. Amounts of endogenous *Mamld1* and *Akr1c18* relative to that of *Gapdh* were analyzed by TaqMan real-time PCR in three independent experiments.

### Statistical analysis

Data are expressed as the mean ± SEM. Statistical differences in mean values between two groups were examined by Student’s *t*-test or Mann-Whitney’s *U*-test, and differences in frequencies were examined by χ^2^ test. *P* values less than 0.05 were considered significant.

## Additional Information

**How to cite this article**: Miyado, M. *et al.* Parturition failure in mice lacking *Mamld1*. *Sci. Rep.* **5**, 14705; doi: 10.1038/srep14705 (2015).

## Supplementary Material

Supplementary Information

## Figures and Tables

**Table 1:** Frequency of delayed parturition.

**Genotype of mother**	**Genotype of father**	**Delayed parturition**^a^	**Statistical significance**^b^
WT	WT	4/21^c^	
*Mamld1* KO	WT	6/11	*p* = 0.040
*Mamld1* KO	*Mamld1* KO	14/24	*p* = 0.027
WT	*Mamld1* KO	2/11^c^	*p* = 0.83

WT: wildtype; KO: knockout.

^a^The denominators indicate the number of pregnant mice, and the numerators are the number of mice with delayed parturition (≥20.5 days post coitum).

^b^The results are compared to that of WT pairs.

^c^The frequency of delayed parturition in WT animals was comparable between this study and previous studies^3^,^24^.

**Table 2:** Serum steroid hormone levels in WT and *Mamld1* KO mice.

	**Genotype**		**Statistical significance**
18.5 days post coitum^a^			
	WT (*n* = 10)	*Mamld1* KO (*n* = 9)	
Progesterone (ng/mL)	10.9 ± 3.6	26.8 ± 2.6	*p* = 0.0014
20α-OHP (ng/mL)	37.0 ± 5.5	20.6 ± 2.1	*p* = 0.041
Testosterone (pg/mL)	214.7 ± 25.8	272.5 ± 43.3	*p* = 0.87
Estradiol (pg/mL)	27.6 ± 3.8	24.8 ± 4.4	*p* = 0.63
20.5 days post coitum^b^			
	WT (*n* = 4)	*Mamld1* KO (*n* = 5)	
Progesterone (ng/mL)	4.9 ± 2.6	23.0 ± 8.8	*p* = 0.050
20α-OHP (ng/mL)	39.4 ± 4.3	26.2 ± 5.8	*p* = 0.13

WT: wildtype; KO: knockout; 20α-OHP: 20α-hydroxyprogesterone.

The results are expressed as the mean ± SEM.

^a^During pregnancy.

^b^WT mice, at 0 or 1 day postpartum; KO mice, during pregnancy.
