# Supplementary material for: Parturition failure in mice lacking Mamld1
Source: Sci Rep. 2015 Oct 5;5:14705. doi: 10.1038/srep14705 (PMC4592954; doi:10.1038/srep14705)
Supplement: Supplementary Information [file srep14705-s1.pdf]

Supplementary Information

Parturition failure in mice lacking *Mamld1*

Mami Miyado[1], Kenji Miyado[2], Momori Katsumi[1], Kazuki Saito[1], Akihiro Nakamura[2], Daizou Shihara[1], Tsutomu Ogata[1,3], and Maki Fukami[1,]*

[1]Department of Molecular Endocrinology, National Research Institute of Child Health and Development, Tokyo 157-8535, Japan;

[2]Department of Reproductive Biology, National Research Institute of Child Health and Development, Tokyo 157-8535, Japan;

[3]Department of Pediatrics, Hamamatsu University School of Medicine, Hamamatsu 431-3192, Japan

Supplementary Table S1. Parturition induction by progesterone receptor antagonist RU486.

Genotype of female mouse	Administration timing	Parturition induction[a]
WT	17.5 dpc	5/5
WT	18.5 dpc	5/5
Mamld1 KO	17.5 dpc	7/7
Mamld1 KO	18.5 dpc	6/6

WT: wildtype; KO: knockout; dpc: days post coitum.

[a] The denominators indicate the number of treated mice, and the numerators represent the number of mice in which vaginal bleeding (the signs of labour initiation) or delivery of the first pup was induced within 24 hours after RU486 administration.

Supplementary Table S2. TaqMan assay kits utilized in this study.

Gene symbol	TaqMan assay ID
Mamld1	Mm01293665_m1
Akr1c18 (*20α-Hsd*)	Mm00506289_m1
Cyp11a1	Mm00490735_m1
Cyp17a1	Mm00484040_m1
Cyp19a1	Mm00484054_m1
Esr1	Mm01191130_m1
Fp (*Ptgfr*)	Mm00436055_m1
Hsd17b1	Mm00501692_g1
Hsd17b3	Mm00515131_m1
Hsd17b7	Mm00501703_m1
Hsd3b2	Mm00462685_m1
Jund	Mm04208316_s1
Lgals3	Mm00802901_m1
Nr4a1	Mm01300401_m1
Notch1	Mm00435249_m1
Notch4	Mm00440525_m1
Otxr	Mm01182684_m1
Prlr	Mm04336676_m1
Socs3	Mm00545913_s1
Star	Mm00441558_m1
Stat5b	Mm00839889_m1
Srd5a1	Mm00614213_m1
Gapdh	4352339E (Mm99999915_g1)

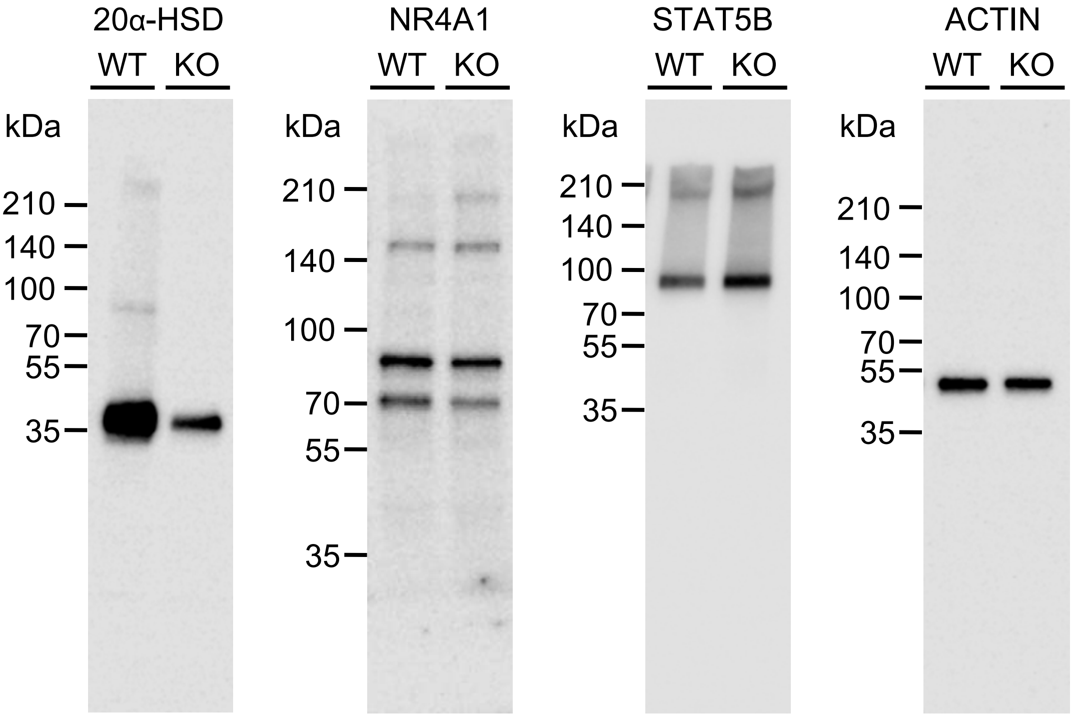

Supplementary Figure S1. Immunoblotting analysis of pregnant mice ovaries.
Western blot analysis of ovaries obtained from wildtype (WT) and *Mamld1* knockout (KO) mice at 18.5 days post coitum. ACTIN was analyzed as an internal control.

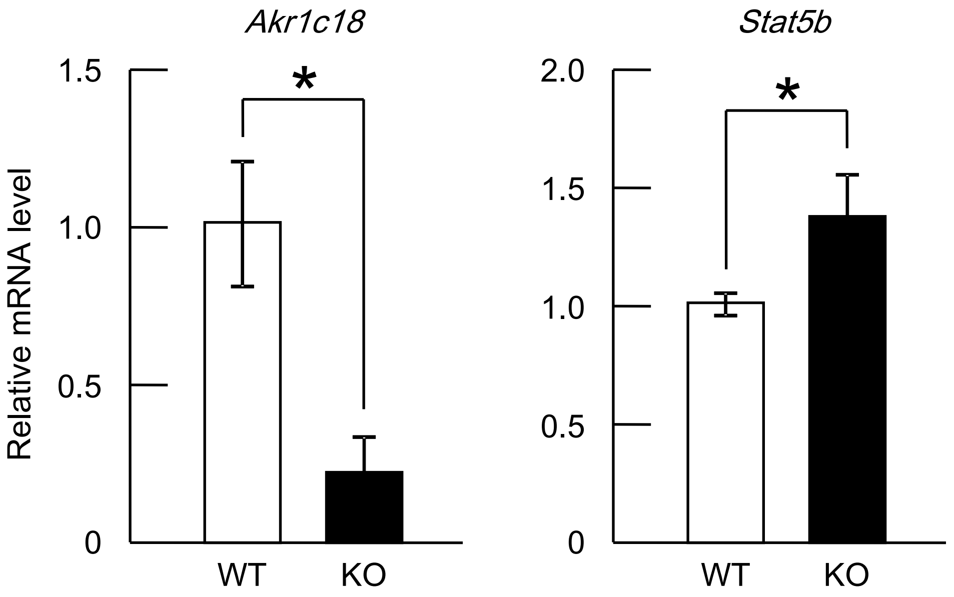

Supplementary Figure S2. Expression patterns of functional luteolysis-related genes in pregnant WT and *Mamld1* KO mice.

Relative mRNA levels of genes in the whole ovaries in non-pregnant WT (at parturition day or one day after parturition, *n* = 4) and pregnant KO (*n* = 5) mice at 20.5 dpc are shown. mRNA levels relative to that of *Gapdh* are shown. The results are expressed as the mean ± SEM. The average of mRNA levels in WT mice was defined as 1.0. Asterisks indicate statistical significance. ACTIN was analyzed as an internal control.
